# Improvement in Left and Right Ventricular Function after Introduction of SGLT2 Inhibitors in Heart Failure Outpatients with Reduced Ejection Fraction

**DOI:** 10.3390/clinpract13060116

**Published:** 2023-10-25

**Authors:** Gianmarco Alcidi, Rosanna Pugliese, Sara Ioannoni, Matteo Romano, Gianpaolo Palmieri, Erika Tabella, Michele Correale, Matteo Di Biase, Natale Daniele Brunetti, Massimo Iacoviello

**Affiliations:** 1 School of Cardiology, Department of Medical and Surgical Sciences, University of Foggia, 71122 Foggia, Italy; gianmarco.alcidi@unifg.it (G.A.); rosanna.pugliese@unifg.it (R.P.); sara.ioannoni@unifg.it (S.I.); matteo.romano@unifg.it (M.R.); gianpaolo.palmieri@unifg.it (G.P.); erika.tabella@unifg.it (E.T.); matteo.dibiase@unifg.it (M.D.B.); natale.brunetti@unifg.it (N.D.B.); 2 Cardiology Unit, University Polyclinic Hospital of Foggia, 71122 Foggia, Italy; michele.correale@libero.it

**Keywords:** heart failure with reduced ejection fraction, type 2 sodium-glucose cotransporter inhibitors, cardiac function, two-dimensional speckle tracking

## Abstract

Background: Type 2 sodium-glucose cotransporter inhibitors (SGLT2i) are among the main therapeutic options for patients with chronic heart failure with reduced ejection fraction (HFrEF). The aim of this study was to evaluate the effects of SGLT2i on the echocardiographic parameters of left (LV) and right (RV) ventricular function among outpatients with a long history of HFrEF, in optimized therapy. Methods: We evaluated consecutive patients affected by HFrEF in whom the SGLT2i therapy was prescribed. Following a baseline evaluation (T0), in which SGLT2i was prescribed, patients were re-evaluated at 3 (T3), 6 (T6), and 12 (T12) months. Results: We considered 60 patients for the analysis with a median history of HFrEF of more than seven years in optimal medical and electrical therapy. After SGLT2i therapy, LV ejection fraction and LV global longitudinal strain improved from baseline at T3, T6, and T12. Analogously, RV global and free wall longitudinal strain improved at T3 and T6. Conclusions: Our study shows that the addition of SGLT2i to the optimized therapy for HFrEF was associated with a significant improvement in both LV and RV function, thus highlighting a possible mechanism responsible for the benefit obtained with this class of drugs.

## 1. Introduction

Sodium-glucose cotransporter type 2 inhibitors (SGLT2i) have been demonstrated to significantly improve the outcome of patients affected by chronic heart failure (CHF) with reduced ejection fraction (HFrEF) [1,2]. They are currently strongly recommended for the treatment of these patients [3]. Notably, despite solid evidence regarding the ability of SGLT2i to improve outcomes, the mechanisms underlying their beneficial effects remain to be fully clarified [4,5,6,7,8,9,10,11,12]. It has been hypothesized that SGLT2i benefits in heart failure as well as in patients affected by type 2 diabetes mellitus (T2DM) or chronic renal disease are related to effects at different levels, i.e., renal [8], metabolic [9,10], and cardiac [11]. Certainly, renal protection could play a prominent role [8]. However, it could also be relevant that the beneficial effect on cardiac function is mediated by direct and indirect effects such as the improvement in myocardial energetics [10,11], a reduced intramyocardial sodium concentration [11], and increased oxygen delivery related to an increase in haemoglobin [12]. However, there are limited data about the effects of SGLT2i on cardiac function in HFrEF patients [13,14,15,16,17].

In a retrospective study with a longer follow-up, an improvement in left ventricular (LV) function was observed, but only in patients affected by T2DM [14]. In diabetic and non-diabetic patients, an improvement in ventricular function was observed, but only during a short-term follow-up [15,16]. Interestingly, this study showed an improvement in LV and right ventricular (RV) function. This is very relevant since there are few data about the efficacy of the current recommended therapy for HFrEF in improving RV function. Consequently, further data are needed to better clarify the effects of SGLT2i on RV besides those on LV function.

This study aimed to evaluate the 12-month effects of SGLT2i on the echocardiographic parameters of both LV and RV function among diabetic and non-diabetic outpatients with a long history of HFrEF and optimized therapy.

## 2. Materials and Methods

The enrolled patients were all referred to the heart failure unit of the University Policlinic Hospital of Foggia for HFrEF. They were selected from the Daunia registry, a single-center observational registry, which also aimed to prospectively evaluate the effects of new therapeutic approaches on clinical and echocardiographic parameters. Local ethics committees approved the registry, and all enrolled patients provided written informed consent.

All patients to whom therapy with SGLT2i was prescribed were considered. Following the requirements of the Italian Ministry of Health, we enrolled patients to whom dapagliflozin (since February 2022) or empagliflozin (since June 2022) could be prescribed and reimbursed by the National Health System, i.e., with New York Heart Association (NYHA) class II–III, left ventricular ejection fraction (LVEF) ≤ 40%, already treated, if not contraindicated or not tolerated, with ACE-inhibitors (ACEi) or angiotensin II receptor blockers (ARBs) or sacubitril/valsartan, mineralocorticoid receptor antagonists (MRA), and beta-blockers [3]. Patients undergoing cardiac resynchronization therapy (CRT) were implanted at least 6 months before enrollment.

Patients with a recent diagnosis of heart failure and those without optimal treatment in the last six months were excluded. Patients with atrial fibrillation and irregular rhythm and those with poor quality echocardiographic imaging were also excluded due to the limitations in assessing ventricular function by two-dimensional speckle tracking (2D-ST) analysis.

Baseline evaluation (T0) was considered the evaluation before SGLT2i were started. Every effort was made to repeat the baseline evaluations at 3 (T3), 6 (T6), and 12 (T12) months. Moreover, evaluations conducted between 6 and 12 months before initiating SGLT2 inhibitor therapy (T-6/12) were analyzed when available.

**Figure 1:**
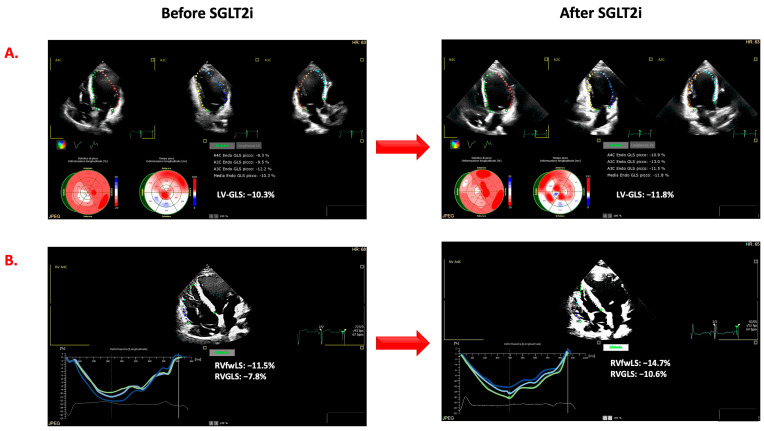
Changes in the two-dimensional strain parameters reflecting left (**Panel A**) and right (**Panel B**) ventricular functions before (**left**) and after (**right**) the onset of type 2 of sodium-glucose cotransporter inhibitors. LV-GLS: left ventricular global longitudinal strain; RV-fwLS: right ventricular free wall longitudinal strain; RV-GLS: right ventricular global longitudinal strain.

At each time, the following evaluations were performed:

- Medical visit and ECG: history of ischemic heart disease, arterial hypertension, diabetes mellitus, estimated glomerular filtration rate (GFR), NYHA class, arterial pressure, and rhythm and heart rate at ECG were recorded. The dose of heart failure classes of drugs was evaluated as follows: for ACEi, the equivalent enalapril dose was calculated according to the following proportions: enalapril 20 mg/day equivalent to ramipril 10 mg/day, zofenopril 30 mg/day, and lisinopril 20 mg/day. For ARBs, the equivalent valsartan dose was calculated according to the following proportions: valsartan 320 mg/day equivalent to losartan 100 mg/day, and candesartan 32 mg/day [3]. For beta-blockers, the equivalent bisoprolol dose was calculated according to the following proportions: bisoprolol 10 mg/day equivalent to carvedilol 50 mg/day, nebivolol 10 mg/day, metoprolol tartrate 200 mg/day. Finally, the sacubitril/valsartan dose of 24/26 mg b.i.d. was computed as 100 mg/day, that of 49/51 mg b.i.d. as 200 mg/day, and that of 97/103 mg b.i.d. as 400 mg/day.
- Echocardiographic examinations. Echocardiographic examinations were analyzed by two operators (G.A., R.P.) who were in the blind from the time of the evaluation as well as from the results of the other examinations of each patient. In accordance with current recommendations, LVEF was calculated on the basis of left ventricular end-diastolic volume (LVEDV) and end-systolic volume (LVESV) (Simpson’s rule). The ratio between E and e′ (E/e′) was based on the peak of the E wave (E) at pulsed Doppler and the TDI peak of early diastolic velocity peak (e′) at the level of the septal and lateral mitral annulus [18]. Mitral (MR) and tricuspid (TR) regurgitation were evaluated and quantified by arbitrary units (a.u. range from 0 to 4). Tricuspid annular plane systolic excursion (TAPSE) was assessed in order to evaluate RV systolic function. As shown in Figure 1, the strain measurements were obtained by the AutoStrain application of the Philips EPIQ CVx ultrasound system. From the “off-cart” analysis of the stored examinations, the LV global longitudinal strain (LV-GLS) was measured by the analysis of standard two-, three-, and four-chamber views and the average values of all segments. Using the RV-focused four-chamber view, the RV function was assessed by automatically calculating the global longitudinal strain of the right ventricle (RV-GLS) and that of the free wall (RV-fwLS). Although the AutoStrain application allows for semiautomatic evaluation, the region of interest, the automatically detected cardiac cycle, and the segmental analyses’ accuracy were verified and corrected when appropriate. Ventricular strain measurements are expressed as negative values, i.e., the lower the value, the better the ventricular function. For this reason, in the manuscript, we indicated more negative values than those determined at baseline as improved ventricular systolic strain. LV reverse remodeling was defined as a reduction of LVESV greater than 15% from baseline [19], whereas a significant improvement in LVEF was defined as an absolute increase of more than 5% from baseline. The improvement in LV-GLS, RV-GLS, and RV-fwLS was defined as a relative change of more than 10% from baseline [20].

Statistical analysis. The continuous variables are expressed as mean values ± SD. Continuous variables were compared by Student’s *t*-test, whereas dichotomized variables by Fisher’s test. In order to assess the relationship between the variables, Pearson’s linear correlations were used. The linear mixed model for repeated measurements was used to test differences among the values observed at each evaluation, i.e., T-6/12, T0, T3, T6, and T12. Statistical analyses were performed using STATA software, Version 12 (StataCorp, College Station, TX, USA) or Statistica 6.1 software (StatSoft Inc., Tulsa, OR, USA). A *p* value of <0.05 was considered statistically significant.

## 3. Results

**Figure 2:**
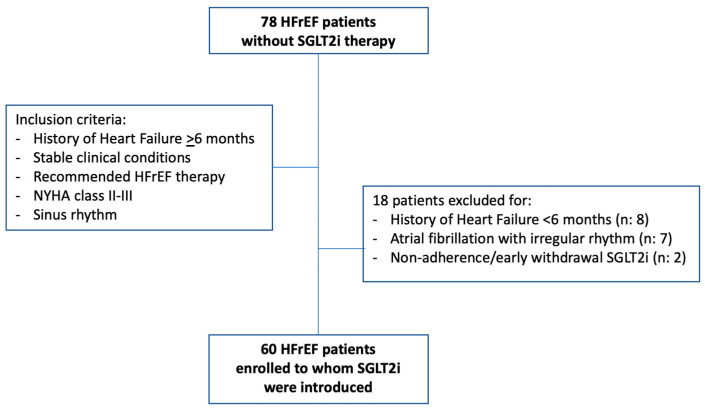
Selection of screened patients. NYHA: New York Heart Association; SGLT2i: type 2 sodiu-glucose cotransporter inhibitors. n: number.

Out of 78 patients, 18 were excluded, nine because they had a recent history of HFrEF, seven because of atrial fibrillation with irregular rhythm, and two because of non-adherence and early withdrawal of SGLT2i therapy (Figure 2). The clinical characteristics of the remaining patients are shown in Table 1. Among these, due to the timing of the National Health System rules on the reimbursement of SGLT2i, dapagliflozin was prescribed in most of the patients (number: 55).

### 3.1. Changes in Echocardiographic Parameters before SGLT2i Introduction

In order to evaluate the changes in the studied echocardiographic variables before the enrollment, for 43 patients, a comparison between baseline evaluation and those available from 6 to 12 months before baseline was performed. No significant differences were found in LVEDV (mean relative change −1.1 ± 14.1%), LVEF (mean relative change −3.2 ± 16.9%), LV-GLS (mean relative change +3.7 ± 20.5%); MR (mean relative change −0.4 ± 36.5%); LAVI (mean relative change +14.9 ± 53.4%); E/e′ (mean relative change +11.2 ± 48.1%); TAPSE (mean relative change −3.9 ± 15.7%); RV-GLS (mean relative change: +8.7 ± 32.1%); RV-fwLS (mean relative change: +7.3 ± 30.3%); and TR (mean relative change: −11.5% ± 37.1%).

### 3.2. Changes in Echocardiographic Parameters after the Introduction of SGLT2i 

All enrolled patients repeated baseline evaluations at least once at 3 months (T3) or 6 months (T6). T3 was available in 53 patients and T6 in 54. In 48 patients, it was also available at 12 months (T12). The serial analysis of LVEF and LV-GLS was available for all patients, whereas RV strain measurements were available in only 53 patients due to the quality of imaging. Table 2 reports the baseline (T0) mean values observed as well as the relative changes from baseline in the analyzed echocardiographic parameters after the introduction of SGLT2it.

**Figure 3:**
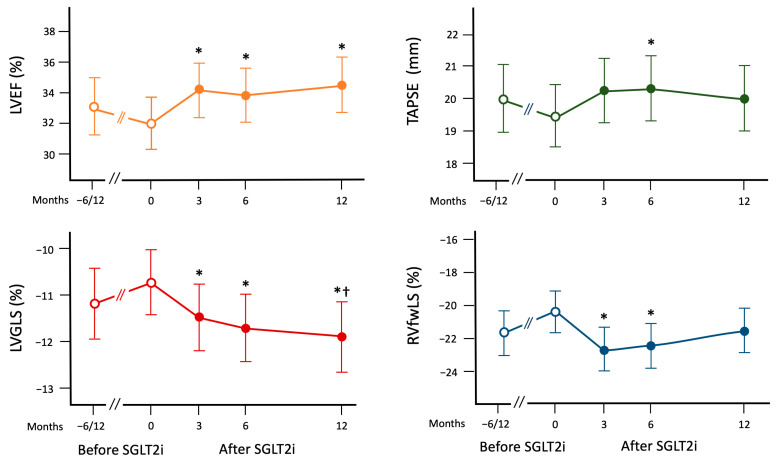
Changes in the parameters reflecting left and right ventricular functions after the onset of type 2 of sodium-glucose cotransporter inhibitors. The data are expressed as mean and 95% confidence interval at linear mixed model for repeated measurements. LVEF: left ventricular ejection fraction; LV-GLS: left ventricular global longitudinal strain; RV-fwLS: right ventricular free wall longitudinal strain; RV-GLS: right ventricular global longitudinal strain. *p* refers to linear mixed model. * *p* < 0.05 vs. T0; † *p* < 0.05 vs. T-6/12.

Figure 3 summarizes the changes in the LVEF, LV-GLS, TAPSE, and RV-fwLS. LVEF significantly improved from baseline at T3, T6, and T12. Analogously, LV-GLS showed a significant improvement from T0 to T3, T6, and T12. Moreover, the improvements at T6 and T12 were significant also when compared to the T6/12 values. When the parameters of RV function were considered, a significant improvement in TAPSE and RV-GLS from baseline was observed at T6 whereas an improvement in RV-GLS and RV-fwLS was observed at T3 and T6. Figure 1 shows the case of a patient with improvement in both LV and RV function assessed by 2D-ST.

When all changes from baseline were analyzed, relative changes in LV-GLS were significantly correlated with relative changes in LVEF (r = 0.592; *p* < 0.001) and relative changes in RV-GLS (r = 0.347; *p* < 0.001), but not with those in RV-fwLS (r = 0.121; *p* = 0.160). Relative changes in RV-GLS were significantly correlated with absolute changes in LVEF (r = 0.334; *p* < 0.001) and relative changes in RV-fwLS (r = 0.617; *p* < 0.001). No correlations were found between changes in LAVI and E/e′ and those observed for LVEF and LV-GLS. Relative changes in E/e′ were significantly correlated with improvements in RV-GLS (r: −0.347; *p* = 0.001) and TAPSE (r: 0.308; *p* = 0.002).

A reverse remodeling, defined as a reduction of LVESV > 15%, was observed in 38% of the enrolled patients whereas an improvement in LVEF was observed in 30%. A greater percentage of patients experienced an improvement in LV-GLS (60%), RV-fwLS (58%), and RV-GLS (60%).

## 4. Discussion

The main finding of this study is that, in diabetic and non-diabetic HFrEF patients receiving the recommended therapy, SGLT2i can improve both RV and LV functions, as assessed by conventional parameters and 2D-ST during a 1-year follow-up. An improvement in both LV and RV function was already observed in previous studies. Indeed, a recent metanalysis demonstrated the relationship between SGLT2i and an improvement in LV, but not in RV function. Mustapic et al. [15,16], in a single-blind randomized study, showed the beneficial effects of SGLT2i on LV and RV function in optimally treated HFrEF patients, but over a short-term follow-up. Over a longer follow-up, the retrospective study of Hwang et al. [14] showed an improvement in LV function, but only in patients affected by T2DM who were not taking sacubitril/valsartan.

Consequently, our study adds new evidence about the possible effects of SGLT2i. We enrolled patients with HFrEF and a long history of heart failure who had already received optimal medical or electrical therapy for at least six months. Notably, 98% of patients were taking beta-blockers, 85% received MRA, 22% underwent CRT, and a significant percentage were on ACEi/ARB and sacubitril/valsartan. These therapies maximize reverse cardiac remodeling and improve LVEF when combined [21,22]. Therefore, the effects of introducing SGLT2i in our series are even more relevant. The administration of SGLT2i led to a rapid improvement in systolic function after just three months, with a stable trend observed at 6 and 12 months. Although we did not have a control group, the relationship between SGLT2i and the observed beneficial effects is further supported by the fact that a large subgroup of patients evaluated 6 to 12 months before starting SGLT2i did not show significant differences compared to their status at the time of enrollment.

These findings further support the hypothesis that SGLT2i exert their effects through complementary mechanisms, with an additive effect when combined with other recommended drugs [3,23]. This outcome is highly relevant, especially considering that despite randomized controlled trials demonstrating the ability of SGLT2i to reduce heart failure hospitalizations across different LVEF values, the underlying mechanisms behind these effects have not been fully clarified. Although the effect of SGLT2i on glomerular hyperfiltration may play a pivotal role in cardiorenal protection [8], other potential direct and indirect effects on cardiac function have been hypothesized, particularly in terms of changes in myocardial energetics [5,9,10]. One such mechanism involves a change in the use of metabolic substrates for energy production [9,10], which could lead to improved myocardial energetics in both ventricles. Additionally, SGLT2i may contribute to reducing cytosolic sodium and calcium levels and increasing mitochondrial calcium, further positively impacting cardiac function [11]. Moreover, increased cardiac oxygen delivery due to elevated hematocrit and afterload reduction could potentially enhance ventricular function in heart failure and diabetic patients without heart failure [12].

These multifaceted effects highlight the complex and promising nature of SGLT2i in heart failure management and can explain the improvements in both LV and RV function that we observed. This last finding is relevant from a pathophysiological and clinical point of view. RV function could play a relevant role in the progression of heart failure. Many studies have demonstrated the independent and incremental prognostic relevance of an impaired RV function. However, as of today, there are no specific therapeutic approaches for enhancing RV function [24]. The improvement in RV function after heart-failure therapy optimization could be related to a direct effect on RV or could be the consequence of LV improvement. A recent meta-analysis evaluated all the studies reporting RV function and pulmonary pressure after introducing sacubitril/valsartan [25]. The results demonstrated that sacubitril/valsartan could improve LV and RV function in HFrEF. However, the improvement in RV function did not seem to be entirely dependent on LV reverse remodeling [25]. Analogously, in our patients, the changes in LV and RV function were weakly correlated, thus suggesting the possibility that the beneficial effects of SGLT2i on RV function could be in part related to an effect that is independent of the improvement in the LV. Future studies need to explore better how the efficacy of SGLT2i in improving outcomes could be mediated by the improvement in one or both ventricular systolic functions. Finally, future studies should further clarify the improvement in RV function over time. In fact, in our series, it is smoothened at twelve months.

The results of our study about the beneficial effects of SGLT2i on both LV and RV systolic function are further reinforced by the analysis of 2D-ST derived parameters. 2D-ST provides information about segmental and global myocardial deformation. In particular, in recent years, longitudinal strain analysis has demonstrated greater sensitivity in detecting early myocardial dysfunction compared to traditional echocardiographic parameters [26]. Moreover, this analysis has shown enhanced accuracy in stratifying the prognosis of heart failure patients by assessing both LV and RV systolic function [27,28]. Furthermore, the semi-automatic analysis of 2D-ST that we employed is less influenced by operators [26], ensuring greater objectivity and reliability in our findings. Altogether, using 2D-ST adds robustness to our observations, providing a more comprehensive assessment of the beneficial cardiac effects resulting from SGLT2i treatment.

In our series, the evaluation of natriuretic peptides was not available for all patients. This is a very relevant point to better clarify the pathophysiological mechanisms underlying the benefits of SGLT2i on LV and RV function. Natriuretic peptides are strongly related to LV remodeling and hemodynamic and neurohormonal status [29]. For this reason, the beneficial effects of sacubitril/valsartan on LV remodeling are strongly related to a drop in natriuretic peptide levels, thus also reflecting the strong effect of this therapeutic strategy on neuro-hormonal status [21]. On the other hand, SGLT2i showed only a modest effect on natriuretic peptide levels [29]. Future studies should address the relationship between the effects of SGLT2i on LV and RV function and changes in natriuretic peptides to understand the mechanisms of the beneficial effects of SGLT2i.

Our study had other several limitations. We enrolled a small group of patients. Although we attempted to minimize the limitations related to an observational study by evaluating patients with a long history of CHF under optimal medical therapy and by serially analyzing changes in systolic parameters in comparison with baseline and previous echocardiographic evaluations, the absence of a control group without SGLT2i represents a significant limitation. A control group would have allowed for a more robust comparison and assessment of the specific effects of SGLT2i treatment. Additionally, it is worth noting that only a small number of patients in our series was affected by diabetes. This disparity is because, in Italy, diabetic patients might have already received therapy with SGLT2i, while this option was available only to non-diabetic patients since the beginning of our enrollment. Therefore, the influence of baseline metabolic status on the response in terms of systolic function should be further investigated in future studies. By acknowledging these limitations, we can better contextualize the results of this study, and future research can build upon these findings to address these gaps in understanding.

## 5. Conclusions

For patients with HFrEF undergoing optimal medical therapy, the administration of SGLT2i was associated with a short to midterm improvement in both LV and RV function. These data provide new evidence about the mechanisms underlying the beneficial effects of this class of drugs, suggesting the direct and indirect effects of SGLT2i. Further studies should confirm these data and better clarify the relationship between RV and LV improvement and the influence of diabetes.

## Figures and Tables

**Table 1:** Patient baseline clinical characteristics.

Variable	
Number	60
Age (years)	62 ± 15
Males, n (%)	48 (80)
Median time from diagnosis of CHF (years)	7
Ischemic etiology, n (%)	27 (45)
Diabetes mellitus, n (%)	13 (22)
Arterial Hypertension, n (%)	35 (58)
NYHA class II, n (%)	46 (75)
III, n (%)	14 (25)
BMI (kg/m^2^)	27 ± 5
SAP (mm Hg)	118 ± 17
Heart rate (beats/minute)	68 ± 11
LVEF (%)	32 ± 6
Creatinine (mg/dL)	1.18 ± 0.34
GFR-EPI (mL/min/1.73 m^2^)	66 ± 22
Concomitant therapy at the enrollment	
Sacubitril/Valsartan, n (% among treated)	41 (68)
Sacubitril/Valsartan > 50% target dose	14 (34)
ACE-I/ARB, n (%)	19 (32)
ACE-I/ARB ≥ 50% target dose, n (% among treated)	4 (21)
Beta-blockers (%)	59 (98)
Beta-blocker ≥ 50% target dose, n (% among treated)	35 (59)
MRA, n (%)	51 (85)
MRA dose ≥ 100% target dose, n (% among treated)	30 (59)
Ivabradine, n (%)	17 (28)
Loop diuretics, n (%)	39 (64)
Furosemide-equivalent dose (mg/day)	54 ± 50
ICD, n (%)	55 (90)
CRT, n (%)	37 (22)

ACE-I: inhibitors of Angiotensin-Converting Enzyme; ARB: angiotensin II receptor blockers; BMI: body mass index; GFR-EPI: estimated glomerular filtration rate by EPI formula; CRT: cardiac resynchronization therapy; ICD: implantable cardioverter-defibrillator; LVEF: left ventricular ejection fraction; MRA: mineralcorticoid receptor antagonists; NYHA class: New York heart Association class; SAP: systolic arterial pressure.

**Table 2:** Baseline mean values and relative changes in the studied parameters before and after type 2 sodium-glucose cotrasporter inhibitor treatment. For ventricular strain measurements, the negative changes correspond to improvement in ventricular function.

	Baseline Values	Relative Changes from T0		
	T0	T3	T6	T12
LVEDV	155 ± 58 mL	−4.7 *±* 14.6%	−3.6 *±* 17.3	−4.4 *±* 17.3
LVEF	32 ± 6%	+8.2 *±* 18.0% *	+8.5 *±* 24.9% *	+10.4 *±* 26.1% *
LV-GLS	−10.7 ± 2.7%	−8.7 *±* 21.1% *	−12.2 *±* 26.0% *	−14.1 *±* 33.2% *
MR	1.4 ± 0.7 a.u.	+1.6 *±* 36.9%	+3.8 *±* 40.8%	+10.3 *±* 45.4%
LAVI	36 ± 18 mL/m2	+5.4 *±* 33.5%	+10.2 *±* 39.1%	+0.1 *±* 33.8%
E/e′	9.6 ± 3.2	+3.3 ± 38%	+3.2 *±* 38%	+17.9 *±* 53.8%
TAPSE	19.5 ± 3.8 mm	+6.4 *±* 19.8%	+4.8 *±* 18.9% *	+4.3 ± 18.2%
RV-GLS	−14.6 ± 3.3%	−15.6 *±* 32.1% *	−9.9 *±* 27.8% *	−11.0 *±* 29.5%
RV-fwLS	−20.4 ± 4.3%	−13.5 *±* 22.6% *	−10.9 *±* 22.3% *	−8.9 *±* 25.7%
TR	1.3 ± 0.6 a.u.	+2.9 *±* 37.6%	−2.1 *±* 37.1%	+5.6 *±* 49.1%

Baseline values expressed as the mean ± standard deviation; relative changes from baseline expressed as mean percentage changes ± standard deviation; * *p* < 0.05 vs. T0; *p* refers to the comparison of mean values by linear mixed models. E/e′: ratio between early diastolic peak at pulsed Doppler and mean value of e′s and e′l; LVEDV: Left ventricular end-diastolic volume; LVEF: left ventricular ejection fraction; LV-GLS: left ventricular global longitudinal strain; MR: mitral regurgitation; LAVI: atrial volume indexed for body surface area; RV-fwLS: right ventricular free wall longitudinal strain; RV-GLS: right ventricular global longitudinal strain; free wall right ventricular longitudinal strain;TAPSE: tricuspid annulus systolic excursion; TR: tricuspid regurgitation.

## Data Availability

The data is available on request.
